# The research capacity and culture of Australian podiatrists

**DOI:** 10.1186/s13047-015-0066-9

**Published:** 2015-03-28

**Authors:** Cylie M Williams, Peter A Lazzarini

**Affiliations:** Peninsula Health, Community Health, PO Box 52, Frankston, VIC 3199 Australia; Monash University, School of Physiotherapy, Frankston, VIC 3199 Australia; School of Clinical Sciences, Queensland University of Technology, Brisbane, QLD 4059 Australia; Allied Health Research Collaborative, Metro North Hospital & Health Service, Queensland Health, Rode Rd, Brisbane, QLD 4032 Australia

**Keywords:** Research, Culture, Capacity, Australia, Podiatry

## Abstract

**Background:**

Best practice clinical health care is widely recognised to be founded on evidence based practice. Enhancing evidence based practice via the rapid translation of new evidence into every day clinical practice is fundamental to the success of health care and in turn health care professions. There is little known about the collective research capacity and culture of the podiatry profession across Australia. Thus, the aim of this study was to investigate the research capacity and culture of the podiatry profession within Australia and determine if there were any differences between podiatrists working in different health sectors and workplaces.

**Method:**

All registered podiatrists were eligible to participate in a cross-sectional online survey. The Australian Podiatry Associations disseminated the survey and all podiatrists were encouraged to distribute it to colleagues. The Research Capacity and Culture (RCC) tool was used to collect all research capacity and culture item variables using a 10-point scale (1 = lowest; 10 = highest). Additional demographic, workplace and health sector data variables were also collected. Mann–Whitney-U, Kruskal–Wallis and logistic regression analyses were used to determine any difference between health sectors and workplaces. Word cloud analysis was used for qualitative responses of individual motivators and barriers to research culture.

**Results:**

There were 232 fully completed surveys (6% of Australian registered podiatrists). Overall respondents reported low success or skills (Median rating < 4) on the majority of individual success or skill items. Podiatrists working in multi-practitioner workplaces reported higher individual success or skills in the majority of items compared with sole practitioners (p < 0.05). Non-clinical and public health sector podiatrists reported significantly higher post-graduate study enrolment or completion, research activity participation, provisions to undertake research and individual success or skill than those working privately.

**Conclusions:**

This study suggests that podiatrists in Australia report similar low levels of research success or skill to those reported in other allied health professions. The workplace setting and health sector seem to play key roles in self reported research success and skills. This is important knowledge for podiatrists and researchers aiming to translate research evidence into clinical practice.

## Background

Research is a major driver of global health care improvements [1-3]. Best practice clinical health care is widely recognised to be founded on evidence based practice [3-5]. Evidence based practice is defined as the integration of the best available evidence from systematic research combined with clinical expertise [3]. Enhancing evidence based practice via the rapid translation of new research evidence into every day clinical practice is fundamental to the success of health care and health professions [2,5,6]. However, the rate at which this success is achieved appears to be underpinned by the evidence based practice and research culture of the health profession [4,5,7]. The evaluation of the collective research culture and skills (or ‘capacity’) of health professions provides a platform for reflection, adaption and growth in this highly evidence based practice health care environment.

The field of research aimed at understanding research capacity and culture in health professions is growing [5,8-10]. Historically, measurement of research capacity and culture has primarily focussed on academic research outputs such as numbers of publications, citations, research higher degree students and research funding in the health profession [8,9]. More contemporary measures are now combining the measurement of these research outputs with the measurement of research inputs, such as self-rated research knowledge and skill tools, to give a more holistic picture of the overall research culture of a profession or organisation [10-13]. The recently developed and validated Research Capacity and Culture tool has become an established method of measuring and benchmarking these research inputs and outputs, particularly in those of the allied health professions working within public health care environments [11,13].

The research capacity and culture of allied health professions has been the focus of many recent studies using a number of different qualitative and quantitative measures [5,7,11,14,15]. Studies indicate that in comparison to the medical and nursing professions, the allied health professions report significantly lower levels of research capacity and culture [9,11,15,16]. Allied health professions report very high levels of interest in research yet they conversely report very low levels of capacity to actually participate in research activities [11,14,16]. A number of common allied health barriers and motivators for undertaking and building research capacity and culture have also been identified within this body of literature [13,14,17]. These barriers consistently include lack of time for research due to increased clinical loads and perceived research skill deficits [13,18,19], while motivators include personal desire to improve skill sets, job satisfaction and increased opportunities for career advancement [13,18,19].

The allied health profession of podiatry has seen rapid growth in Australia since the change from professional certification to undergraduate university qualifications in 1977 [20]. There are now over 4,000 podiatrists registered in Australia; a 74% increase over the last decade [21]. The podiatry profession has a growing public and academic sector yet the vast majority of the podiatry workforce in Australia is employed within clinical roles in the private sector with many working as sole practitioners [21,22]. At an undergraduate level there is a strong evidence based practice teaching commitment by the universities, however, it appears that there are limited post-graduate opportunities for podiatrists.

The podiatry profession has displayed a very positive attitude to participating in research in the past [14,19,23]. Yet like other allied health professions, there appears to be low baseline skills in many of the areas needed to undertake research activities as reported in a small study of Queensland public sector podiatrists [14,19,24]. There is little known about the collective research capacity and culture of the podiatry profession across Australia [19]. It is not known if the sector or workplace in which a podiatrist practices influences the research capacity and culture [19]. In order for the podiatry profession to maximise its impact on health outcomes into the future and ensure evidence guides clinical practice it is essential to understand the research capacity and culture of the profession [19]. Podiatrists in Australia work within the private, public and education settings in a variety of clinical, education, administrative and research role within these various health sectors and workplaces. In 2012, it was estimated that 69% of podiatrists primary workplace was within a private setting, 16% within a community based health services, 9% within hospitals and almost 2% within education facilities. There were 95% of podiatrists working within clinical roles, 2.5% of podiatrists primarily working in administration roles and 0.7% in research roles, however it is not specifically known what setting podiatrists with administration role or research roles work in (I.e. A private practice owner only working the administration side of the business or the podiatrist working in research within the hospital setting) [25].

The primary aim of this study was to investigate the research capacity and culture of the podiatry profession within Australia. The secondary aim was to determine if there were any differences in the research capacity and culture of podiatrists working in different health sectors and workplaces.

## Method

### Study design

This study was a cross-sectional survey.

### Participants and setting

Eligible participants were all registered podiatrists (n = 4017) within Australia [26]. The survey was disseminated by email flyers, newsletters and online media through the Australian Podiatry Council, and state based Australian Podiatry Associations.

### Measurements

All participant data were collected via the one electronic survey. The survey had two overarching components containing general demographics variables and research capacity and culture variables. General demographic variables were collected from each participant including gender, age group, Australian state of practice, original podiatry qualification, university that granted original qualification, status of any further post-graduate study, years post qualification, size of organisation and primary work roles. Participants were also requested to identify the workplace they practiced: sole or multi-practitioner (for those who worked in the same organisation or team with other podiatrists or health care practitioners); and; the type of health sector in which they primarily practiced (>50% of time): private, public community, public hospital, non-clinical (including education, research and/or administrative roles).

The Research Capacity and Culture (RCC) tool was used to collect all research capacity and culture variables [11]. This tool has demonstrated acceptable reliability and validity to measure the numerous indicators which influence research culture within Australian public health settings [11]. The RCC contains 51 items examining self-reported success or skill in a range of areas related to research capacity or culture at the individual, team or organisational level. The tool uses a 10 point scale with one being the lowest and 10 being the highest skill level possible. The tool also gives participants the opportunity to record their perceived barriers and motivators to research in both closed and open ended questions. To reduce missing data, a forced response was used throughout the questionnaire and the “don’t know” option was removed.

### Procedure

Following dissemination of the survey link, each participant gave consent and completed the survey online. The survey was open from the 11^th^ of February 2014 to the 4^th^ of May, 2014. There was monthly advertising of the survey via the same modalities as outlined in the original survey dissemination above and all podiatrists were encouraged to share the advertisement with fellow podiatrists.

The responses were collected using Qualtrics online survey software [27] and utilised skip logic to capture information from both sole practitioners and podiatrists who worked with other podiatrists or health care professionals. Skip logic ensured that the participant only was asked questions relevant to their workplace (e.g. sole practitioners were not asked to self rate organisation and team questions) The survey also was set to ensure full completion at each section with the participant not being able to continue the survey without full completion of that previous section. The participants were able to withdraw from the survey at any time by closing the browser and any non-completed questions were treated as missing data for the remaining non-completed variables.

The Human Research Ethics Committee of Peninsula Health, Victoria, Australia, approved this study (LRR13PH27).

### Analysis

Data were analysed using Stata SE [28]. Descriptive statistics were used to express each demographic variable in numbers and percentages. The RCC results were analysed as ordinal categorical data and median and interquartile ranges recorded due to non-normal distribution of responses. Mann Whitney U analysis was used to compare the scores between the sole practitioner and multi-practitioner workplaces. Kruskal–Wallis one-way analysis of variance was used to compare any differences between subgroups of health sectors. Logistic regression was used to analyse the individual responses across the four binary data outcomes (Yes/No) for activities, provisions, motivators and barriers against the different workplaces with. A minimum significance level of p < 0.05 was used. A complete case analysis approach without imputation of missing data was undertaken. Open text questions were analysed and represented with word cloud methodology with NVivo Version 10 [29]. With word cloud analysis the grammatical and non-frequent words are hidden and those words of greater frequency are displayed in larger font. The statements from participants were separated into team enablers and team barriers.

## Results

There were 397 surveys initiated (10% of Australian registered podiatrists as of 2014). There was a drop out of 158 participants at the first question asking the self rating of success or skill. One participant dropped out between the team and individual self ratings and a further six participants dropped out at the demographics portion of the survey leaving a total of 232 fully completed surveys (6% of Australian registered podiatrists as of 2014). Table 1 gives a breakdown of the demographics of participants (listing participant numbers at each question to account for missing data from non-completion due to early exit of survey). Table 1 also gives the demographic breakdown based on the primary health sector of practice subgroup: private (n = 131 with n = 94 working 100% of their time in this sector), public community (n = 45 with n = 15 working 100% of their time in this sector), public hospital (n = 28 with n = 12 working 100% of their time in this sector) and non-clinical, including managers, educators and academics (n = 28 with n = 6 working 100% of their time in this sector). There were 39% (n = 90) of participants working across two sectors and 5% (n = 12) of participants working across 3 or more sectors. The distribution of podiatrists that responded was compared to the 2012 HWA Report and was similar for the private sector (55% vs 69%), public community (20% vs 16%) and public hospital (9% vs 12%). No comparison was able between the non-clinical and published data due to data collection methods. There were a number of podiatrists who had both completed and initiated more than one post-graduate degree. There were significant differences between the private setting, two public settings and non-clinical health sector subgroups with regards to research-related activities being part of the job role (p < 0.001).

**Table 1:** **Demographics of participant items – number of responses (n) and percentage of podiatrist responses (%)**

	**Total responses**	**Private**	**Public community**	**Public hospital**	**Non-clinical**
	**(232 podiatrist)**	**(131 podiatrists)**	**(45 podiatrists)**	**(28 podiatrists)**	**(28 podiatrists)**
	**N (%)**	**n (%)**	**n (%)**	**n (%)**	**n (%)**
**Gender**					
*Male*	74 (32)	47 (36)	11 (24)	5 (18)	11 (39)
*Female*	153 (67)	84 (64)	32 (71)	23 (82)	17 (61)
*Declined to answer*	2 (1)	0 (0)	2 (4)	0	0 (0)
**Age**					
*<25*	16 (7)	12 (9)	2 (4)	1 (4)	1 (4)
*25-29*	50(21)	22 (17)	14 (31)	9 (32)	5 (18)
*30-34*	36(16)	17 (13)	10 (22)	4 (14)	5 (18)
*35-39*	28 (12)	15 (11)	4 (9)	3 (11)	6 (21)
*40-44*	29 (13)	14(11)	7 (16)	3 (11)	5 (18)
*45-49*	29 (13)	20 (15)	2 (4)	4 (14)	3 (11)
*50-59*	40 (17)	30 (23)	5 (11)	3 (11)	2 (7)
*60-69*	4 (1)	1 (1)	1 (2)	1 (4)	1 (4)
**State/s of practice**					
*Queensland*	54 (23)	33 (25)	9 (20)	4 (14)	8 (29)
*Northern Territory*	2 (1)	2 (1)	0 (0)	0 (0)	0 (0)
*Western Australia*	18 (8)	11 (8)	2 (4)	3 (11)	2 (7)
*Australian Capital Territory*	1 (1)	1 (1)	0 (0)	0 (0)	0 (0)
*New South Wales*	24 (10)	20 (15)	0 (0)	0 (0)	4 (14)
*Victoria*	98 (42)	44 (33)	28 (62)	17 (61)	9 (32)
*South Australia*	30 (13)	18 (13)	5 (11)	3 (11)	4 (14)
*Tasmania*	10 (4)	6 (4)	2 (4)	1 (4)	1 (4)
**Podiatry degree**					
*Queensland University of Technology*	37 (16)	24 (18)	5 (11)	2 (7)	6 (21)
*La Trobe University*	82 (35)	32 (24)	27 (60)	15 (54)	8 (29)
*Charles Sturt University*	4 (2)	3 (2)	1 (2)	0 (0)	0 (0)
*Curtin University of Technology*	13 (6)	8 (6)	1 (2)	2 (2)	2 (7)
*Overseas (Please list)*	22 (10)	11 (80)	5 (11)	3 (3)	3 (11)
*University of South Australia*	27 (12)	18 (14)	3 (7)	2 (2)	4 (14)
*University of Newcastle*	5 (2)	4 (3)	0 (0)	0 (0)	1 (4)
*Other Australian University not listed*	2 (1)	2 (2)	0 (0)	0 (0)	0 (0)
*Sydney Technical College*	6 (3)	6 (5)	0 (0)	0 (0)	0 (0)
*Sydney Institute of Technology*	7 (3)	6 (5)	0 (0)	0 (0)	1 (4)
*Western Australian Institute of Technology*	0 (0)	0 (0)	0 (0)	0 (0)	0 (0)
*University of Western Australia*	5 (2)	3 (2)	1 (2)	1 (4)	0 (0)
*University of Western Sydney*	2 (1)	0 (0)	0 (1)	0 (0)	2 (7)
*Lincoln Institute of Health Sciences*	11 (5)	7 (5)	1 (2)	3 (11)	0 (0)
*South Australian Institute of Technology*	9 (4)	7 (5)	1 (2)	0 (0)	1 (4)
**Original qualification**					
*Certificate*	3 (1)	3 (2)	0 (0)	0 (0)	0 (0)
*Associate Diploma*	6 (3)	6 (5)	0 (0)	0 (0)	0 (0)
*Diploma*	46 (21)	31 (24)	5 (11)	3 (11)	7 (25)
*Bachelor*	166 (72)	85 (65)	37 (82)	23 (82)	21 (75)
*Masters*	10 (4)	6 (5)	2 (4)	2 (7)	0 (0)
*Doctorate*	1 (1%)	0 (0)	1 (2)	0 (0)	0 (0)
**Undertaking further study**					
*Undertaking a Masters (by research or coursework) degree*	32 (14)	8 (6)	10 (22)	10 (36)	4 (12)
*Undertaking a PhD*	10 (4)	1 (1)	1 (2)	0 (0)	8 (24)
*Undertaking a Clinical Doctorate*	2 (1)	2 (2)	0 (0)	0 (0)	0 (0)
*Completed a Masters (Research) degree*	10 (4)	6 (5)	1 (2)	1 (1)	2 (6)
*Completed a Masters (Clinical) degree*	19 (8)	6 (5)	3 (7)	5 (18)	5 (15)
*Completed a PhD*	9 (4)	3 (2)	0 (0)	1 (4)	5 (15)
*Completed a Clinical Doctorate*	0 (0)	0 (0)	0 (0)	0 (0)	0 (0)
*No further study*	164 (71)	110 (84)	32 (71)	13 (46)	9 (27)
**Years of practice (full time equivalence)**					
*0-5 years*	61(26)	36 (27)	16 (36)	7 (25)	2 (7)
*6-10 years*	46 (20)	21 (16)	10 (22)	9 (32)	6 (21)
*11-15 years*	29 (13)	17 (13)	6 (13)	1 (4)	5 (18)
*>15 years*	96 (41)	57 (44)	13 (29)	11 (39)	15 (54)
**Hours of work**					
Part Time (<35 hours)	78 (34)	47 (36)	13 (29)	7 (25)	11 (39)
Full time (>35 hours)	154 (66)	84 (64)	32 (71)	21 (75)	17 (61)
**Workplace**					
*Sole practitioner*	66 (29)	57 (43)	6 (13)	1 (4)	2 (7)
*2-4 podiatrists*	93 (40)	56 (43)	19 (42)	7 (25)	11 (39)
*5-10 podiatrists*	49 (21)	17 (13)	14 (31)	12 (43)	6 (21)
*11 or more podiatrists*	24 (10)	1 (1)	6 (13)	8 (29)	9 (32)
**Size of organization**					
*Sole practitioner*	43 (19)	41 (18)	2 (4)	0 (0)	0 (0)
*<10*	87 (38)	72 (38)	4 (9)	1 (4)	10 (36)
*11-100*	29 (13)	13 (12)	13 (29)	0 (0)	3 (11)
*101-1000*	37 (15)	4 (16)	21 (47)	6 (21)	6 (21)
*>1000*	36 (15)	1 (1)	5 (11)	21 (75)	9 (32)
**Primary work role (up to 2 choices)**					
*Patient/client podiatry service provision*	214 (93)	131 (100)	44 (98)	25 (89)	14 (50)
*Supervision or mentor of other podiatrists*	41 (18)	19 (15)	6 (13)	9 (32)	7 (25)
*Manager/team leader of other podiatrists*	39 (17)	19 (15)	2 (4)	8 (25)	10 (36)
*Administration (includes research/education)*	24 (10)	7 (5)	2 (4)	2 (7)	13 (46)
**Research related activities part of job role**					
No	160 (69)	115 (88)	27 (60)	13 (46)	5 (18)
Yes	70 (30)	15 (11)	17 (38)	15 (54)	23 (82)
Not applicable	2 (1)	1 (1)	1 (2)	0 (0)	0 (0)

Table 2 displays the median ratings and interquartile range results, for the sections pertaining to organisation and team research success or skills, from the 158 participants who worked in multi-practitioner workplaces. Half (9 of 18) of the items pertaining to organisation research skills, and 10 of 19 pertaining to team research skills recorded median scores of less than adequate (<5). Team research culture was described by participants as being influenced by the organisation direction and the organisational commitment to research activities. Many participants identified that organisational priorities, funding and time were the main barriers and some participants stated, “*The organisation appears to be only focused on seeing clients*” and “*as private practitioners our goal is treatment not research*”. Participants identified team cohesiveness also influenced culture citing barriers to starting a research project as “*getting everyone in the one room to keep it all coordinated*” and “*specific identified areas the whole team are happy with*”. Many team research cultures motivators were also identified and the team research outputs contribution to evidence based practices was the primary motivator reported. Participants also identified that specific workers motivated their teams such as managers, team leaders and research leads within the organisation to undertake research activities. Organisation support and promotion of staff engagement within research activity was reported by a number of participants, all of who worked within the public sector in community health or acute. The acknowledgement of the podiatry team’s importance within the organisation was also identified within motivators to undertake research projects. Participants stated that the “recognition of team” and “organisational recognition” together with “age differences between practitioners and difference in treatment techniques” being motivators and promoting a positive team culture.

**Table 2:** **Organisation and team research skill statement items for all participants in multi-practitioner workplaces**

**Organisation research skill statement**	**N**	**median (IQR)**
*1. Has adequate resources to support staff research training*	158	6 (3–8)
*2. Has funds, equipment or admin to support research activities*	158	4 (2–7)
*3. Has a plan or policy for research development*	158	3 (1–7)
*4. Has senior managers that support research*	158	6.5 (3–9)
*5. Ensures staff career pathways are available in research*	158	3 (1–7)
*6. Ensures organisation planning is guided by evidence*	158	7 (5–9)
*7. Has consumers involved in research*	158	4 (1–7)
*8. Accesses external funding for research*	158	2 (1–7)
*9. Promotes clinical practice based on evidence*	158	8 (7–9)
*10. Encourages research activities relevant to practice*	158	7 (3–8)
*11. Has software programs for analysing research data*	158	3 (1–7)
*12. Has mechanisms to monitor research quality*	158	2.5 (1–6)
*13. Has identified experts accessible for research advice*	158	5 (2–8)
*14. Supports a multi-disciplinary approach to research*	158	6 (2–8)
*15. Has regular forums/bulletins to present research findings*	158	4 (2–8)
*16. Engages external partners (eg universities) in research*	158	4 (1–8)
*17. Supports applications for research scholarships/ degrees*	158	6 (2–8)
*18. Supports the peer-reviewed publication of research*	158	6 (2–9)
Team research skill statement		
*1. Has adequate resources to support staff research training*	158	4 (2–7)
*2. Has funds, equipment or admin to support research activities*	158	3 (1–6)
*3. Does team level planning for research development*	158	3 (1–7)
*4. Ensures staff involvement in developing that plan*	158	4 (1–7)
*5. Has team leaders that support research*	158	5 (2–8)
*6. Provides opportunities to get involved in research*	158	5 (1–8)
*7. Does planning that is guided by evidence*	158	6 (3–8)
*8. Has consumer involvement in research activities/planning*	158	2.5 (1–6)
*9. Has applied for external funding for research*	158	2 (1–6)
*10. Conducts research activities relevant to practice*	158	4 (1–8)
*11. Supports applications for research scholarships/ degrees*	158	5 (1–8)
*12. Has mechanisms to monitor research quality*	158	4 (1–7)
*13. Has identified experts accessible for research advice*	158	5 (1–8)
*14. Disseminates research results at research forums/seminars*	158	5 (1–8)
*15. Supports a multi-disciplinary approach to research*	158	6 (2–8)
*16. Has incentives & support for mentoring activities*	158	5 (1–7)
*17. Has external partners (eg universities) engaged in research*	158	3 (1–8)
*18. Supports peer-reviewed publication of research*	158	6 (2–8)
*19. Has software available to support research activities*	158	3 (1–7)

**Figure 1:**
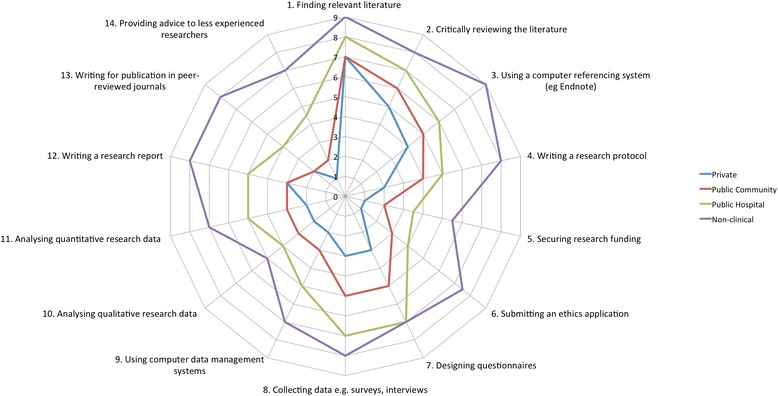
**Differences in individuals based on primary health sector of employment (medians shown).**

Table 3 reports the median ratings and interquartile range results, for the sections pertaining to individual research skills, for all participants and the workplace subgroups. Participants working in multi-practitioner workplaces (n = 157) recorded significantly higher individual skill levels in all items compared with those working as sole practitioners (n = 81) (p < 0.01). Table 4 reports the results of individual research skill for the primary health sector of practice subgroups. Median scores increased in all individual research skill items for each health sector from private, public community, public hospital, to non-clinical sector subgroups as illustrated in Figure 1 (p < 0.01).

**Table 3:** **Individual research skill statement items for all participants and workplace subgroups (p)**

**Individual research skills**	**All Podiatrists**	**Sole practitioner**	**Multi-practitioner**	
	**N = 238**	**N = 81**	**N = 157**	
	**Median (IQR)**	**Median (IQR)**	**Median (IQR)**	**p**
1. Finding relevant literature	7 (6–8)	6 (5–8)	7 (6–9)	0.003
2. Critically reviewing the literature	6 (4–8)	5 (4–7)	7 (5–8)	0.001
3. Using a computer referencing system (eg Endnote)	5 (2–8)	3 (1–6)	6 (2–8)	0.001
4. Writing a research protocol	3 (1–6)	1 (1–4)	4 (2–7)	<0.001
5. Securing research funding	1 (1–4)	1 (1–2)	2 (1–5)	0.001
6. Submitting an ethics application	2 (1–5)	1 (1–2)	2 (1–7)	<0.001
7. Designing questionnaires	4 (1–7)	2 (1–5)	5 (2–7)	<0.001
8. Collecting data e.g. surveys, interviews	5 (2–7)	3 (1–5)	6 (2–8)	<0.001
9. Using computer data management systems	3 (1–6)	2 (1–4)	4 (2–7)	0.004
10. Analysing qualitative research data	3 (1–5)	1 (1–3)	3 (1–6)	<0.001
11. Analysing quantitative research data	3 (1–6)	2 (1–4)	4 (2–7)	0.001
12. Writing a research report	3 (1–7)	2 (1–5)	5 (2–7)	<0.001
13. Writing for publication in peer-reviewed journals	2 (1–6)	1 (1–4)	3 (1–7)	<0.001
14. Providing advice to less experienced researchers	2 (1–6)	1 (1–2)	3 (1–6)	<0.001

**Table 4:** **Individuals research skill statement items for all participants and health sector subgroups**

**Individual research skills**	All Podiatrists*	**Private**	**Public community**	**Public hospital**	**Non-clinical**	
	**n = 238**	**n = 131**	**n = 45**	**n = 22**	**n = 28**	
	**median (IQR)**	**median (IQR)**	**median (IQR)**	**median (IQR)**	**median (IQR)**	***X*** ^***2***^ **(DF), p**
1. Finding relevant literature	7 (6–8)	7 (5–8)	7(5–8)	8 (7–9)	9 (7.5-9)	26.16 (3), <0.001
2. Critically reviewing the literature	6 (4–8)	5 (4–7)	6 (5–8)	7 (6–8)	8 (7–9)	25.37 (3), <0.001
3. Using a computer referencing system (eg Endnote)	5 (2–8)	4 (1–7)	5 (2–7)	6 (4.5-8)	9 (5–9)	26.24 (3), <0.001
4. Writing a research protocol	3 (1–6)	2 (1–5)	4 (1–5)	5 (2–8)	8 (5.5-9)	37.64 (3), <0.001
5. Securing research funding	1 (1–4)	1 (1–2)	2 (1–4)	3.5 (1–6.5)	5.5 (2.5-7)	55.46 (3), <0.001
6. Submitting an ethics application	2 (1–5)	1 (1–2)	3 (1–5)	4 (1–7.5)	7.5 (4–9)	48.71 (3), <0.001
7. Designing questionnaires	4 (1–7)	3 (1–6)	5 (3–7)	7 (5–8)	7 (5–9)	34.54 (3), <0.001
8. Collecting data e.g. surveys, interviews	5 (2–7)	3 (1–6)	5 (3–7)	7 (6–8)	8 (7–9)	49.96 (3), <0.001
9. Using computer data management systems	3 (1–6)	2 (1–5)	3 (2–5)	5 (2–7.5)	7 (5–8)	34.96 (3), <0.001
10. Analysing qualitative research data	3 (1–5)	2 (1–4)	3 (1–5)	4 (1–6)	5 (2–7)	17.80 (3), 0.001
11. Analysing quantitative research data	3 (1–6)	2 (1–5)	3 (1–6)	5 (2–7)	7(4.5-8)	27.48, (3), <0.001
12. Writing a research report	3 (1–7)	3 (1–5)	3 (1–6)	5 (5–7.5)	8 (5–8.5)	27.97 (3), <0.001
13. Writing for publication in peer-reviewed journals	2 (1–6)	2 (1–4)	2 (1–5)	4 (1–7)	8 (5–9)	35.69 (3), <0.001
14. Providing advice to less experienced researchers	2 (1–6)	1 (1–4)	2 (1–5)	4.5 (2–6.5)	7 (5–8)	40.35 (3), <0.001

*Total participants completed the individual research skills however only 226 participants identified a workplace.

Table 5 displays the current research activity and research provisions reported for all participants and the primary health sector subgroups. Overall, the majority of participants (60%) recorded no current participation in research activities. Data collection (21%) or writing research reports, presentations or publications (18%) were the most common research activities reported. The non-clinical subgroup reported significantly more research activity in all items, except the ‘other’ item, than other health sector subgroups (p < 0.01). These results were also reflected in the provisions for research items reported where the non-clinical subgroup also reported significantly more provisions for research that other health sector subgroups (p < 0.01) in Table 5. Overall, many participants (45%) recorded they had no provisions for research, however, the next most common responses were that participants had computer access (42%) and library access (24%) to assist in research activity. Again the non-clinical subgroup reported significantly more total provisions for research with an average of 5.21 provisions per podiatrist compared to an average of 1.20 provision for the private sector podiatrists (p = 0.034).

**Table 5:** **Current individual research activities and provisions for research items for all participants and health sector subgroups**

**Activity**	**All podiatrists**	**Private**	**Public community**	**Public hospital**	**Non-clinical**		
	**(n = 238)**	**(n = 131)**	**(n = 45)**	**(n = 28)**	**(n = 28)**		
	**n (%)**	**n (%)**	**n (%)**	**n(%)**	**n (%)**	**p**	**OR, 95% CI**
Not currently involved with research	143 (60)	108 (82)	20 (44)	12 (43)	4 (14)	<0.001	0.33, 0.24-0.45
Collecting data eg surveys, interviews	49 (21)	8 (6)	11 (24)	10 (36)	18 (64)	<0.001	2.73, 1.99-3.73
Writing a research report, presentation or paper for publication	44 (18)	9 (7)	5 (11)	8 (28)	21 (75)	<0.001	3.12, 2.22-4.39
Analysing quantitative research data	28 (12)	4 (3)	2 (4)	5 (18)	16 (57)	<0.001	3.57, 2.33-5.49
Writing a literature review	27 (11)	6 (5)	5 (11)	3 (11)	13 (46)	<0.001	2.40, 1.67-3.45
Submitting an ethics application	27 (11)	3 (2)	3 (7)	5 (18)	16 (57)	<0.001	3.76, 2.42-5.84
Writing a research protocol	23 (9)	3 (2)	1 (2)	3 (11)	16 (57)	<0.001	4.28, 2.57-7.13
Applying for research funding	16 (7)	0 (0)	1 (2)	3 (11)	12 (43)	<0.001	6.87, 2.99-15.75
Analysing qualitative research data	15 (6)	2 (2)	2 (4)	5 (18)	5 (18)	0.001	2.35, 1.46-3.80
Other (Including – mentoring, clinical audits, post graduate studies including research, ethics committee representative.	12 (3)	7 (5)	8 (18)	1 (4)	3 (11)	0.480	1.16, 0.77-1.75
Provisions for research							
No provisions	106 (45)	83 (63)	12 (27)	11 (39)	0 (0)	<0.001	0.35, 0.25-0.50
Access to computers	101 (42)	31 (24)	28 (63)	15 (54)	27(96)	<0.001	2.63, 1.94-3.57
Library access	57 (24)	5 (4)	15 (33)	16 (57)	21 (75)	<0.001	3.75, 2.65-5.32
Time	41 (17)	10 (8)	9 (20)	9 (32)	13 (46)	<0.001	2.09, 1.54-2.83
Access to research experts	32 (13)	2 (2)	9 (20)	8 (29)	13 (46)	<0.001	2.91, 2.02-4.19
Software	30 (13)	7 (5)	4 (9)	3 (11)	16 (57)	<0.001	2.66, 1.85-3.81
Research supervision	26 (11)	3 (2)	5 (11)	6 (21)	12 (43)	<0.001	2.86, 1.93-4.23
Research equipment (e.g. digital recorders)	23 (10)	2 (2)	3 (7)	4 (14)	14 (50)	<0.001	3.78, 2.38-6.07
Training	21 (9)	0 (0)	5 (11)	6 (21)	10 (36)	<0.001	3.39, 2.12-5.40
Administrative support	20 (8)	7 (5)	3 (7)	2 (7)	8 (3)	0.003	1.79, 1.22-2.64
Research funds	15 (6)	2 (2)	2 (4)	2 (7)	9 (32)	<0.001	3.05, 1.81-5.12
Other	9 (4)	5 (4)	1 (2)	0 (0)	3 (11)	0.439	1.26, 0.71-2.22
Average number of provisions per podiatrist	2.02	1.20	2.13	2.93	5.21	0.034	1.00, 0.13-1.32

**Figure 2:**
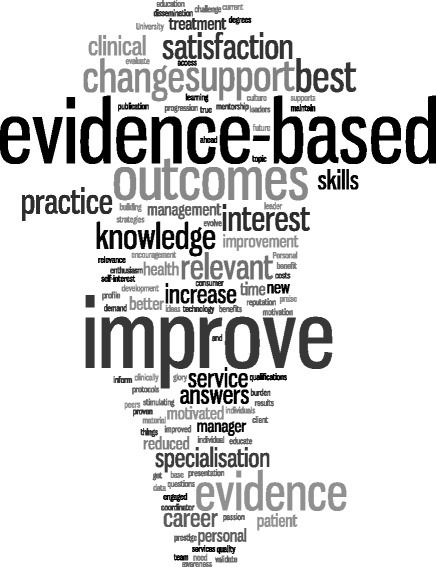
**Word Cloud analysis of word frequency for the motivators of participants in multi-practitioner workplaces.**

**Figure 3:**
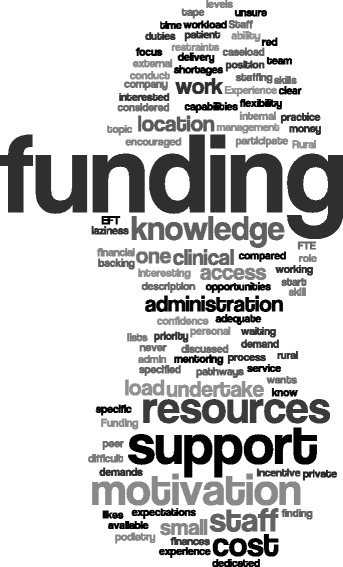
**Word Cloud analysis of word frequency for the barriers of participants in multi-practitioner workplaces.**

Table 6 reported the individual motivators and barriers to undertaking research recorded by participants. Many of these linked with the themes identified by participants working in team environments (Figure 2). Skill development (63%) and increased job satisfaction (51%) were the top two individual motivators, whilst other work taking priority (66%) and lack of time (51%) were the greatest individual barriers. However, there were significant differences in the proportions of responses between health sector subgroups for the majority of motivator and barriers items recorded (p < 0.05). The barriers and motivators to undertake research for those podiatrists working in multi-practitioner environments were visually analysed using word clouds as illustrated in Figures 2 and 3. Words frequently used within the motivators were “evidence-based” “improve” and “outcomes” while “funding”, “resources” and “support” were frequently used in the team barriers.

**Table 6:** **Individual research motivators and barriers items for all participants and health sector subgroups**

**Motivators**	**All podiatrists (n = 238)**	**Private (n = 131)**	**Public community (n = 45)**	**Public hospital (n = 28)**	**Non-clinical (n = 28)**		
	**n (%)**	**N (%)**	**n (%)**	**n(%)**	**n (%)**	**p**	**OR, 95% CI**
To develop skills	147 (63)	70 (53)	30 (67)	26 (93)	21 (75)	0.002	1.52, 1.15-2.02
Increased job satisfaction	122 (51)	50 (38)	28 (62)	23 (81)	21 (75)	<0.001	1.82, 1.38-2.40
To keep the brain stimulated	114 (48)	57 (44)	25 (56)	16 (57)	16 (57)	0.198	1.17, 0.92-1.49
Desire to prove a theory/hunch	93 (39)	51 (39)	15 (33)	14 (50)	13 (46)	0.541	1.08, 0.85-1.38
Problem identified that needs changing	90 (38)	32 (24)	20 (44)	16 (57)	16 (57)	0.002	1.49, 1.16-1.91
Career advancement	82 (34)	26 (20)	19 (42)	20 (71)	17 (61)	<0.001	2.00, 1.53-2.61
Increased credibility	80 (34)	36 (27)	15 (33)	16 (57)	13 (46)	0.012	1.37, 1.07-1.76
Mentors available to supervise	54 (23)	21 (16)	12 (27)	8 (29)	13 (46)	0.002	1.54, 1.17-2.02
Opportunities to participate at own level	48 (20)	26 (20)	9 (20)	9 (32)	6 (21)	0.438	1.12, 0.84-1.49
Forms part of post graduate study	46 (19)	15 (11)	12 (27)	14 (50)	5 (18)	0.012	1.44, 1.09-1.91
Links to universities	42 (18)	17 (13)	8 (18)	8 (29)	9 (32)	0.013	1.45, 1.08-1.94
Colleagues doing research	39 (16)	12 (9)	10 (22)	6 (21)	11 (39)	0.001	1.72, 1.27-2.31
Dedicated time for research	36 (15)	12 (9)	8 (18)	5 (18)	11 (39)	0.001	1.71, 1.26-2.32
Study or research scholarships available	30 (13)	11 (8)	6 (13)	4 (14)	9 (32)	0.005	1.60, 1.16-2.22
No motivators	26 (11)	25 (19)	1 (2)	0 (0)	0 (0)	<0.001	0.08, 0.01-0.57
Research encouraged by managers	25 (11)	6 (5)	8 (18)	4 (14)	7 (25)	0.003	1.73, 1.22-2.45
Research written into role description	24 (10)	4 (3)	9 (20)	3 (11)	8 (29)	0.001	1.93, 1.35-2.78
Grant funds	19 (8)	9 (7)	3 (7)	2 (7)	5 (18)	0.181	1.32, 0.89-1.97
Other	10 (4)	8 (6)	1 (2)	0 (0)	1 (4)	0.163	0.60, 0.27-1.35
Barriers							
Other work roles take priority	158(66)	72 (55)	31 (69)	26 (93)	20(71)	0.001	0.61, 0.45-0.81
Lack of time for research	147 (51)	86 (66)	27 (60)	18 (64)	16 (57)	0.168	0.83, 0.66-1.08
Desire for work/life balance	120 (50)	82 (63)	19 (42)	11 (39)	8 (29)	<0.001	0.58, 0.44-0.75
Lack of skills for research	107 (45)	67 (51)	22 (49)	12 (39)	6 (21)	0.002	0.68, 0.52-0.88
Lack of funds for research	105 (44)	50 (38)	19 (42)	17 (61)	19 (68)	0.005	1.42, 1.11-1.82
Other personal commitments	87 (37)	61 (47)	13 (29)	8 (29)	5 (18)	0.001	0.61, 0.45-0.81
Lack of administrative support	74 (31)	31 (24)	19 (42)	11 (39)	13 (36)	0.016	1.36, 1.06-1.75
Lack of software for research	65 (27)	35 (27)	16 (36)	10 (36)	4 (14)	0.421	0.89, 0.68-1.18
Lack of suitable backfill	61 (26)	17 (13)	17 (38)	15 (54)	12 (43)	0.001	1.80, 1.38-2.35
Lack access to equipment for research	60 (25)	31 (24)	15 (33)	9 (32)	5 (18)	0.809	0.97, 0.73-1.27
Intimidated by fear of getting it wrong	59 (25)	34 (26)	8 (18)	12 (43)	5 (18)	0.789	0.96, 0.73-1.27
Intimidated by research language	57 (24)	30 (23)	8 (18)	8 (29)	3 (11)	0.037	0.73, 0.53-1.00
Not interested in research	55 (23)	47 (36)	2 (4)	2 (7)	4 (14)	<0.001	0.46, 0.31-0.70
Isolation	52 (22)	35 (27)	10 (22)	5 (18)	2 (7)	0.009	0.65, 0.46-0.92
Lack of a co-ordinated approach to research	42 (18)	21 (16)	8 (18)	8 (29)	5 (18)	0.513	1.11, 0.82-1.50
Lack of support from management	50 (21)	22 (17)	11 (18)	11(39)	6(21)	0.164	1.22, 0.92-1.61
Other	15 (6)	12 (9)	0 (0)	2 (7)	1 (4)	0.152	0.66, 0.36-1.23
Lack of library/internet access	12 (5)	7 (5)	3 (7)	1 (4)	1 (4)	0.584	0.85, 0.48-1.53
No barriers	3 (1)	1 (1)	1 (2)	0 (0)	1 (4)	0.441	1.46, 0.57-3.72

## Discussion

This is the largest study undertaken to investigate the research capacity and culture of the podiatry profession within Australia. It also appears to be one of the first nation-wide studies to investigate an allied health profession across different health sectors in Australia. Overall, podiatrists identified success or skills in undertaking early phase research activities such as finding and critically reviewing relevant literature. However, podiatrists reported limited success or skills in later phases of research projects such as the analysis of data or writing for publication. Those working in multi-practitioner workplaces reported their organisation encouraged undertaking research activities. Yet in contrast these podiatrists also reported low levels of resourcing support provided by their organisations for research plans, funding and equipment to actually do this. Subgroup findings suggested that those working in multi-practitioner workplaces and those in the public sector or non-clinical roles reported consistently higher individual research skill levels than their counterparts working in sole practices or private sectors respectively. All items relating to the promotion and use of evidence based practice were rated highly by podiatrists responding to this survey.

The self reported individual research success or skill results were comparable with other allied health professions studied in Australia [11,14,18,30-32] and a smaller study of podiatrists [19]. Studies of dieticians [30], speech pathologists [18], occupational therapists [31], social workers [32], podiatrists [19] and combined allied health professionals [11,14] all reported higher levels of success in early stage research activities yet lower skills in later stage research activities, many of which are necessary for translation of evidence such as writing for publication [11,14,18,19,31,32]. A similar study of Queensland dieticians was the exception to these findings and reported appropriate collective research skills to write a research protocol, submit an ethics application, design questionnaires, use computer data management systems, and write a research report [30]. As podiatry academics were included within this study and dietician academics were also included, it is possible that these participants inflated individual skill or success in the rating of later stage research activities.

While comparable Australian allied health discipline studies have primarily focused on skill sets within the public sector [18,19,30-32] this study of podiatrists appears to be the only allied health study to investigate research culture and capacity within different private, public and non-clinical health sectors in Australia. The inclusion of all these health sector subgroups may be the reason for slightly lower overall collective individual skill levels reported by podiatrists in this study when compared to the small study of public sector podiatrists [19] and those of other allied health disciplines [11,14,18,30-32].

The workplace and health sector in which podiatrists primarily work appears to have a large bearing on the individual research skill and success levels reported. Those podiatrists employed in multi-practitioner workplaces consistently reported higher skill levels to participate in research activities than those working in sole practitioner workplaces. Furthermore there appeared to be escalating individual research skill levels reported in the different health sectors. Non-clinical health sector podiatrists (including research, education and management) reported higher individual research skill levels than those reported in all other health sector subgroups, whilst public hospital podiatrists consistently reported higher individual skill levels than their public community health sector colleagues, and private health sector podiatrists reported the lowest level of individual research skill.

Perhaps unsurprisingly, there appears to be a corresponding relationship between the health sectors with regard to higher proportions of podiatrists who had reported undertaking some form of post-graduate study and/or had research incorporated in their job role and those reporting higher levels of individual research skill and/or higher current research activity participation. Those working in non-clinical health sectors reported enrolment or completion of more post-graduate study, having more research activities incorporated within their job roles, higher research skill levels and more current research activity participation. Conversely, those working in the private sector reported undertaking the least post-graduate study, having the least research activities incorporated in their job roles, the lowest research skill levels and the least current research activity participation. These associations between post-graduate study, research activities in job roles, research skill and research activity participation have also been reported in other allied health discipline studies [18,30]. These factors appear to have significant impacts on the differences in research culture reported between the health sectors. Key differences in motivators and barriers were also apparent between health sectors. The private health sector understandably did not see undertaking research activities as core business. The public health care sectors often have organisation and team plans of which research may be a small or large component. This in turn would impact on individual capacity and culture. It appears more podiatrists within public and non-clinical sectors are expected to undertake research and report greater organisation support to undertake the research in terms of senior management, funding, equipment and time [19]. A recent study of public sector physiotherapy departments found practical ways that a positive research culture is fostered including establishment of research registries, dedicated positions or protected time and regular forums to disseminate research outcomes within the department [33]. This may be a way that podiatrists within the public sector also engage in research activities either as a single profession or within the collective allied health profession and may be the reason that higher ratings of skill and success were seen found. Similarly, capacity building initiatives aimed at increasing team research skills have been demonstrated to also have positive impact on the individual capacity and culture [34]. These results highlight that improving research capacity and culture is multifaceted and that positive changes require initiatives at all levels to improve skills and success. It is hypothesised that the public sector environment enables the podiatrist to be exposed to more research opportunities that in turn would improve their individual research skill sets.

The major barriers and motivators for individuals undertaking research that were identified within this study cohort were also very similar to those identified by other podiatry and allied health studies [13,19]. The major barriers identified were other work roles taking priority, and lack of time, skills and general resources to undertake research [13,19], whereas the common motivators to undertake research were to develop skills, increase job satisfaction, keep the brain stimulated, a problem needs changing, career advancement and increase credibility [13,19]. While the major barriers seem to be consistent across allied health professions, there appear to be a motivator unique to the podiatrist studies and that is the motivator of increased credibility [13,19]. It is postulated that as one of the smaller and relatively newer allied health professions, there may be a sense of a need to prove credibility in the podiatry profession that is not present in the larger established allied health professions [22].

The average number of provisions for research activity differed between each subgroup with the non-clinical subgroup having the highest number of provisions and the private sector had the lowest. Access to research items such as computers, library, training and experts appear to be more commonplace in non-clinical sectors compared to other sectors. This may be presumed essential to the non-clinical workload whereas these may be ancillary to the private sector podiatrist. These disparities in provisions to research should be considered when encouraging a team research capacity building for podiatrists in particular.

There were a number of key limitations with this study. Firstly, the low response rate of 6% of all registered podiatrists suggests that it may be difficult to generalise these results to the rest of the profession. However, due to the method of dissemination the authors were unable to accurately determine the actual denominator population of how many podiatrists were aware of the survey and thus used the largest and most conservative denominator of the entire profession. Furthermore, the cohort of podiatrists responding to this survey appears to be largely similar in terms of median age (35–39 years), female gender (67%) and state of practice proportions (except for New South Wales) to that reported for the broader Australian podiatry population [26]. Recently published workforce data is available within Australia from 2012 was used to compare the response rate of each workplace setting to actual numbers, therefore it is unknown if these are representative of each setting in 2014. Given the relatively stable health sector in each state at the time of survey, it is proposed that these figures may be relatively similar but caution should be used in using total results given the unequal size of subgroups. Given the Grouping managerial, administrative and academic into a “non-clinical” subgroup may not be representative of these potentially heterogeneous roles. The removal of the Unsure/Don’t know option within the RCC may also have impacted on the self reported skills and success together with it’s unprecedented use with private sector podiatrist. Caution in interpretation of findings should be considered due to the subtle change in RCC survey use employed by this study. The use of internet surveys are increasingly being utilised in research, yet this also opens a bias of self selection or non-representative nature of responses. This has the potential to again limit the generalisability of the results to the whole profession. Lastly, the cross-sectional design of this study means it is unable to ascertain a cause-and-effect relationships of variable and can only hypothesise as to the most likely causes for results.

Whilst these limitations need to be taken into consideration, the similarity of these results to other podiatry and allied health professions results in this area, the authors suggest the generalizability of findings of this study may be a better representation of the profession than the low response rate indicates. It appears that the workplace and health sector in which podiatrists practice influence self reported research capacity and culture. It is recommended that future longitudinal research capacity and culture studies investigate the relationships between workplaces and health sectors; with more defined subgrouping of podiatrists to enable further analysis between clinical, managerial, educator and academic podiatrists. With recent changes in undergraduate degree structures and the introduction of mandatory continuing professional development in Australia, there is the real possibility that there will be a shifting emphasis to a more robust research culture within the podiatry profession in the coming years. These results should prompt mindfulness of educators of the level of the current podiatry profession’s research capacity and culture when delivering content, particularly when there is a focus on the translation of evidence. To optimise the reported high level of evidence based practice interest and ensure the long-term successful growth of podiatry profession in Australia the authors support previous recommendations encouraging national approaches to building research capacity and culture in podiatry [19,23].

## Conclusion

This is the first Australian wide study documenting the research culture of podiatry across all health care sectors. This study suggests that the workplace and health sector setting plays a key role in the research skills of individual podiatrists. This is important knowledge for podiatrists, educators, researchers and national professional bodies aiming to rapidly translate research evidence into clinical practice to benefit the podiatry profession and importantly its patients.
